# Echocardiographic Myocardial Work: A Novel Method to Assess Left Ventricular Function in Patients with Coronary Artery Disease and Diabetes Mellitus

**DOI:** 10.3390/medicina60020199

**Published:** 2024-01-24

**Authors:** Alexandra-Cătălina Frișan, Cristian Mornoș, Mihai-Andrei Lazăr, Raluca Șoșdean, Simina Crișan, Ioana Ionac, Constantin-Tudor Luca

**Affiliations:** 1 Cardiology Department, “Victor Babes” University of Medicine and Pharmacy, 2 Eftimie Murgu Sq., 300041 Timisoara, Romania; alexandra.frisan@umft.ro (A.-C.F.); sosdean.raluca@umft.ro (R.Ș.); urseanusimina@yahoo.com (S.C.); ioana_ionac@yahoo.com (I.I.); constantin.luca@umft.ro (C.-T.L.); 2 Institute of Cardiovascular Diseases Timisoara, 13A Gheorghe Adam Street, 300310 Timisoara, Romania; 3 Research Center of the Institute of Cardiovascular Diseases Timisoara, 13A Gheorghe Adam Street, 300310 Timisoara, Romania

**Keywords:** myocardial work, pressure-strain-loop, myocardial dysfunction, left ventricular ejection fraction, global longitudinal strain, coronary artery disease, diabetes mellitus

## Abstract

Myocardial ischemia caused by coronary artery disease (CAD) and the presence of metabolic abnormalities and microvascular impairments detected in patients with diabetes mellitus (DM) are a common cause of left ventricular (LV) dysfunction. Transthoracic echocardiography is the most-used, non-invasive imaging method for the assessment of myocardial contractility. The accurate evaluation of LV function is crucial for identifying patients who are at high risk or may have worse outcomes. Myocardial work (MW) is emerging as an alternative tool for the evaluation of LV systolic function, providing additional information on cardiac performance when compared to conventional parameters such as left ventricular ejection fraction (LVEF) and global longitudinal strain (GLS) because it incorporates deformation and load into its analysis. The potential of MW in various conditions is promising and it has gained increased attention. However, larger studies are necessary to further investigate its role and application before giving an answer to the question of whether it can have widespread implementation into clinical practice. The aim of this review is to summarize the actual knowledge of MW for the analysis of LV dysfunction caused by myocardial ischemia and hyperglycemia.

## 1. Introduction

Coronary artery disease (CAD) is one of the primary causes of death worldwide, mostly caused by atherosclerosis. Over the last four decades, a decrease in the mortality rate caused by CAD has been observed. However, it still accounts for about one third of deaths in individuals over the age of 35. Almost half of the reduction in mortality is due to the upgraded management of acute coronary syndromes (ACS) which encompasses improved prevention and therapeutic strategies [1]. Management of CAD includes the quantitative, accurate and reproducible evaluation of ventricular function. Early detection and treatment of ventricular dysfunction equals a chance to improve the outcome and prognosis of patients with ischemic heart disease. Type 2 diabetes mellitus (DM) is a major risk factor for cardiovascular diseases, and diabetic patients’ risk for heart failure development is two times higher as compared to nondiabetic patients. Moreover, patients with DM have worse cardiovascular outcomes and poorer prognoses [2]. Echocardiography is an essential tool in clinical practice for the evaluation of cardiac function, providing diagnostic and prognostic information in several clinical settings. Left ventricular ejection fraction (LVEF) and global longitudinal strain (GLS) are the most-used echocardiographic parameters for the assessment of left ventricular (LV) systolic function, but their dependence on loading conditions is a major limitation that could lead to the misinterpretation of myocardial contractility. Recently, myocardial work (MW) based on two-dimensional speckle tracking echocardiography has been proposed as a method for assessing myocardial function through the integration of myocardial deformation and afterload, thereby offsetting the disadvantages of conventional parameters. This review summarizes the assessment, the clinical significance and the limitations of myocardial work parameters and outlines their applicability in patients with CAD and in diabetic patients.

## 2. Principles of Myocardial Work

In physics, work is equal to force times distance, and it represents the amount of energy produced to develop a force on an object, a force that is able to affect the object’s position or its amount of movement. Regarding cardiology, cardiac work (stroke work) equals force: it is represented by mean aortic pressure times the distance, that is, stroke volume, and it represents the energy required to eject a volume of blood across the vasculature. The pressure-volume loop, first described by Otto Frank in 1985 [3], gives an insight into cardiac energetics and mechanics. The graphical representation relies on the four phases of the cardiac cycle (passive filling, isovolumetric contraction, ejection and isovolumetric relaxation). The width of the loop represents the difference between the end-diastolic volume and the end-systolic volume, which is defined as stroke volume, and the area within the loop reflects the ventricular stroke work. Cardiac minute work is stroke work multiplied by heart rate, and it directly corelates with myocardial oxygen consumption. The pressure-volume loop is influenced by preload, afterload and the inotropic state of the heart. Also, myocardial ischemia, caused by significant coronary stenosis or spasm, induces focal hypo-contractility and dyssynchrony that disturbs the normal isovolumetric process. Clinical pressure-volume acquisition is an invasive and time-consuming method; thus, it was not implemented into daily clinical practice. Echocardiographic techniques have been developed in the last several years, including volume and strain analysis; therefore, non-invasive estimation of systolic and diastolic pressure-volume relationships became possible [4]. In 2012, Russel et al. introduced a new method for assessing MW non-invasively, based on segmental strain curves obtained by speckle tracking echocardiography combined with an estimated LV pressure curve and using systolic cuff pressure. This method shows good correlation to invasive measurements [5]. While the pressure-volume loop describes the global MW and function, regional MW has been studied in the form of pressure-length loops. Delhaas et al. [6] demonstrated that, similar to the ability of pressure-volume loop to reflect the overall myocardial oxygen consumption, the assessment of the stress-fiber strain area at a regional level can not only reflect regional myocardial work but also reflect the regional oxygen demand. In accordance with this finding, non-invasive pressure-strain loops were shown to reflect regional myocardial oxygen consumption and metabolism, a finding that was validated by 18F-fluorodeoxyglucose positron emission tomography [5]. The ratio of the mechanical energy imparted by the myocardium to the outgoing blood to the total energy consumption is dependent on the loading conditions. An increase in afterload forces the myocardium to maintain the cardiac output against elevated resistance, resulting in the progressive thickening of the left ventricle wall. Initially, the contractile function may remain preserved, but it deteriorates over time, leading to a decline in GLS [7]. MW incorporates afterload as well as regional oxygen consumption and glucose metabolism; therefore, it provides a more comprehensive assessment of myocardial function as compared to deformation parameters alone, such as LVEF and GLS.

## 3. Practical Assessment of Myocardial Work

The initial step in MW acquisition is represented by the noninvasive measurement of blood pressure in the arm, using a sphygmomanometer, while the patient is in the same position as the one used during image acquisition (left lateral recumbent position). This step is crucial because the measured systolic blood pressure is used as an estimate for peak LV pressure in the calculation of MW [8].

The next step is obtaining the three standard transthoracic apical views, with the patient in the left lateral recumbent position. To obtain the best possible image quality, a frame rate of >40 frames/s is necessary to visualize the borders of the myocardium and to minimize the variations in heart rate between the images. At least three consecutive cardiac cycles should be acquired for each apical view. Also, is important to avoid the foreshortening of the apical views, which may lead to the overestimation of myocardial work [9]. The recordings require off-line analysis using a dedicated software (like EchoPAC, version 203, GE Healthcare, Horten, Norway), which allows a semi-automated analysis of speckle-based strain.

Automated functional imaging (AFI) is used to calculate GLS in apical views. A bull’s-eye plot is generated from this analysis and the GLS values are displayed.

The myocardial work button moves to the next step for the MW evaluation. It requires introducing the previously measured blood pressure and then assessing the timing of valvular events (mitral and aortic valve closure and opening), using the apical three-chamber view, by placing a cursor corresponding to the mitral valve closure and opening and the aortic valve closure and opening along the RR interval of the accompanying electrocardiogram trace. The valvular event times can also be set by pulse-wave Doppler recordings at the mitral valve and aortic valve levels in cases where visualizing the valve timings is challenging [7].

**Figure 1:**
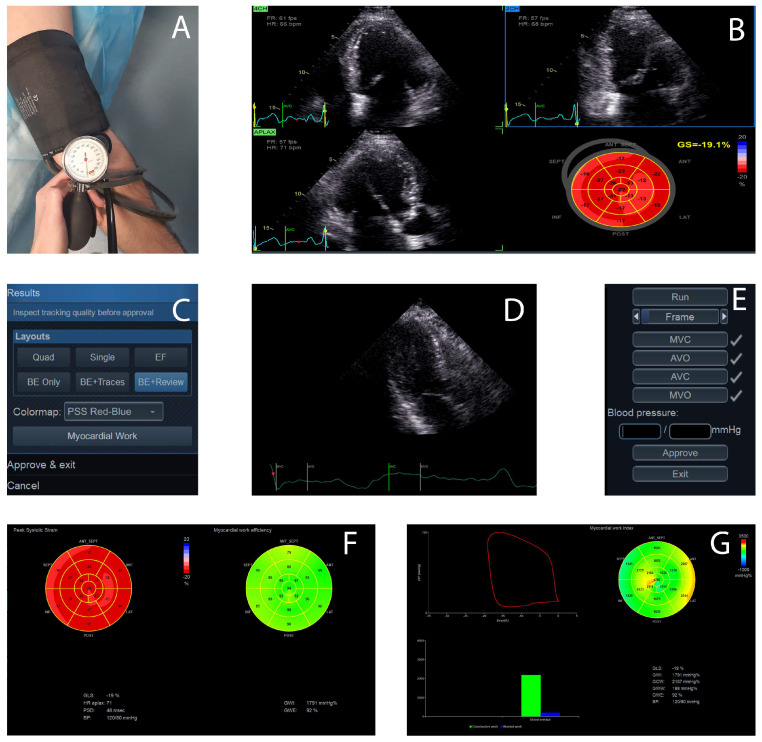
Myocardial work assessment steps. (**A**) *Step one:* blood pressure measurement with an arm cuff. (**B**) *Step two:* two-dimensional grey-scale images acquired in apical two-, three- and four-chamber views, global longitudinal strain and left ventricle bull’s-eye evaluation. (**C**) The myocardial work button moves to the next step for myocardial work evaluation. (**D**) *Step three:* assessment of valvular time events in an apical three-chamber view or by pulsed-wave Doppler recordings at the aortic valve and mitral valve level. (**E**) *Step four:* input of the previous blood pressure measurement. (**F**) *Step five:* visualization of the regional and global myocardial work index and the myocardial work efficiency in bull’s-eye plots. (**G**) *Step six:* analyzation of the pressure-strain loop and additional parameters (global constructive work, global wasted work).

**Figure 2:**
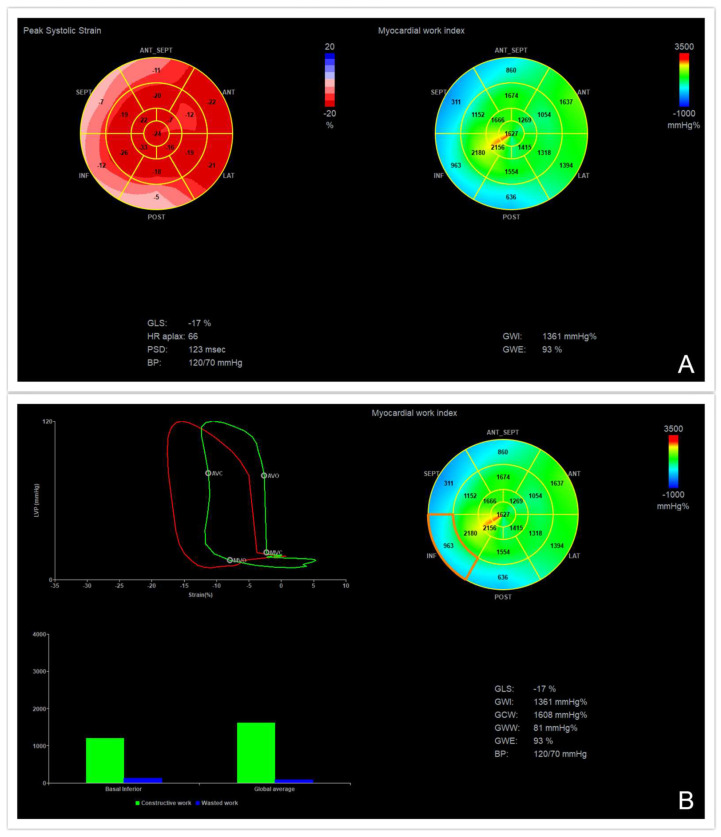
Myocardial work parameters in a patient with an inferior ST−segment elevation myocardial infarction. (**A**) Left ventricular global longitudinal strain, global work index and global work efficiency demonstrating reduced work in the territories supplied by the obstructed right coronary artery. (**B**) Segmental analysis of the inferior wall demonstrating an abnormal pressure−strain loop of the affected myocardium.

After performing all of these steps, the software provides non-invasive pressure strain loops by synchronizing the longitudinal strain, the blood pressure and data about the time of the valvular events previously obtained as well as a bull’s-eye with segmental and global MW values (Figure 1 and Figure 2).

The myocardial work indices are as follows:

GWI (global work index, mmHg%) is the indexed total work within the area of the LV pressure-strain loop, from the mitral valve closure to the mitral valve opening; it corresponds to the myocardial energy translated into mechanical energy throughout the entire systole, including the isovolumic contraction and the isovolumic relaxation [10,11].

GCW (global constructive work, mmHg%) represents the positive myocardial work performed by the LV segments during systolic shortening plus the negative work performed during the lengthening in the isovolumic diastole; it represents the work that contributes to the LV ejection and quantifies the energy consumed by the myocardium that effectively contributes to cardiac output [10,11].

GWW (global wasted work, mmHg%) refers to the negative work performed during the myocardial lengthening in the systole, adding the work performed during the shortening in the isovolumic diastole; this quantifies the energy consumed by the myocardium that is wasted and does not contribute to cardiac output [10,11].

MWE (myocardial work efficiency, %) is defined by the ratio between GCW and the sum of GCW and the GWW; it represents the percentage of myocardial work performed that is translated into cardiac output [10,11].

Global values are determined as the average of all segmental values. The green color of the myocardial work bull’s eye indicates normal values; areas of negative work are represented in blue, while red shows areas of high work [10,11,12].

## 4. Normal Ranges of Myocardial Work Parameters

Manganaro et al. [13] published the first study that established echocardiographic reference ranges for normal, non-invasive MW indices (EACVI NORRE study, 2019) and it was a prospective, multicenter, large-cohort study. A total of 226 heathy subjects (141 women–mean age 44 ± 13 years) from 22 centers were included, and MW analyses were performed and analyzed according to age and gender. GWI and GCW increased with the women’s age, along with systolic and diastolic blood pressure, but after adjustment for confounders, there was no association according to age. GWW and GWE differed only in the subgroup of 20–40 years old when age and gender were considered, with higher values in men than in women. The differences between the genders are related to the effect of blood pressure, which was higher in males. An increase in systolic blood pressure leads to an increase in afterload, resulting in a higher level of energy that shifts the LV work. This study highlighted the association between GWI and GCW with systolic blood pressure and the absence of a strong dependence between MW and age or gender.

One year later, another study aimed to establish reference values for MW indices (STAAB study, 2020) [14] based on a population of apparently healthy individuals from Wurzburg, Germany (779 individuals, 59% women, 49 ± 10 years). GCW, GWE and GWW were independent of sex, and their values were stable up to the age of 45 years. GCW and GWI values were slightly increased in participants around the age of 45 years, without major changes associated with advancing age. The findings could potentially be associated with variations in hormonal status and consecutive changes in blood pressure. In contrast, there was a gradual increase in GWW beyond the age of 45, which is likely attributed to the physiological process of healthy ageing (progressive fibrosis and modulation of cardiomyocytes). Consecutively, GWE decreased with advancing age. Higher blood pressure levels were associated with higher GCW, GWW and GWI, resulting in lower GWE. The study also showed that GCW and GWI correlated with echocardiographic parameters of LV systolic function (LVEF, GLS), while diastolic function (E/e’, left atrium volume) was correlated with GWW. Both systolic and diastolic function were correlated with GWE.

Galli et al. [15] included 115 healthy volunteers in their study (18–69 years, 67% males) to provide the reference values for myocardial work that accounted for age, gender and LV territory. The values of GWI and GCW were higher in women than in men, while no significant difference was observed regarding age. These findings could be explained by the fact that, compared to men, women tend to have higher values of GLS and E-wave velocity, which are the main parameters associated with GWI and GCW. The study also demonstrated a significant difference between LV segmental work from apex to base (GWI, GCW and GWE values were lower in the left ventricular basal segments than in apex, while GWW was lower in the apex).

Recently, Olsen et al. [16] published a prospective study (from the Copenhagen City Heart Study, 2022) that included the general population from Denmark at a large scale (1827 participants, median age 45 years old, 39% men) and reported reference values for myocardial work parameters. Their results showed significant differences between the sexes (GWI, GCW and GWW were higher in women, while GWE was lower) and increasing age for all MW indices. The differences between the sexes may be partially explained by the higher GLS observed in women [17] and by the effect of menopause, which is characterized by declining estrogen levels that influence the cardiovascular system. Furthermore, they observed that abnormal MW indices become increasingly frequent with higher clinical risk (assessed by the Framingham risk score). The normal ranges of MW parameters are listed in Table 1.

## 5. Myocardial Work in Coronary Artery Disease

Cardiovascular diseases, which resulted in 20.5 million deaths in 2021, account for nearly one-third of all global deaths [18]. Ischemic heart disease is the leading cause of premature death worldwide. The advances made in cardiovascular medicine during the last 50 years decreased the globally age-standardized death rate, but the world is far from achieving the goals of diagnosis, treatment, prevention and management of cardiovascular diseases [19]. CAD involves the formation of atherosclerotic plaques in the lumen of coronary arteries, leading to a demand-supply mismatch of oxygen to the myocardium [20]. Non-invasive detection of early ischemia is challenging and is still being investigated. Two-dimensional speckle-tracking echocardiography has been demonstrated to detect early subclinical dysfunction, and it also provides detailed information about global and segmental LV systolic function even when the resting LV wall motion and LVEF are preserved. However, strain parameters remain load-dependent [21]. Recently, a new method that considers loading conditions was validated as being able to non-invasively assess the LV systolic function using longitudinal strain and a standardized LV pressure curve for determining myocardial work. While the loading conditions of LV may not be affected by CAD, the impaired oxygen metabolism in ischemic myocardium segments can have an impact on MW. Accordingly, several studies in the last five years have aimed to investigate the role of MW in patients with CAD. In the following section, we sought to review the latest research publication that evaluates MW in the assessment and prognosis of CAD.

### 5.1. Chronic Coronary Syndromes

To prevent adverse cardiac outcomes, early detection and treatment in patients with significant coronary artery stenosis is very important. LVEF is preserved at rest in most patients with significant CAD, and regional wall-motion abnormalities may not be seen in the early stages of the disease. GLS, assessed by speckle-tracking echocardiography, provides an incremental diagnostic value in detecting CAD in patients with normal LVEF and the absence of regional wall-motion abnormalities [21]. GLS is reduced in the areas of the myocardium affected by ischemia, but an increase in afterload was also demonstrated to be associated with a reduction in LV longitudinal strain. In consequence, in patients with increased afterload conditions (e.g., hypertension), the evaluation of strain may lead to false interpretations of ischemia [22]. Taking afterload into account, MW may be superior to GLS in detecting the myocardial dysfunction caused by CAD.

An interesting study by Edwards et al. [23] included 114 patients referred to angiography who had preserved EF (LVEF ≥55%), no resting regional wall-motion abnormalities and no clinical symptoms of ischemia; this study evaluated whether MW can predict significant CAD. The results showed that global MW was reduced in patients with significant CAD as compared to those without CAD. A receiver operating characteristic (ROC) analysis demonstrated that MW was superior to GLS and was the most powerful predictor of significant CAD. The cutoff value for global MW in predicting CAD was 1810 mmHg% (sensitivity: 92% and specificity: 51%). The low specificity underlines the fact that MW should be used along with other parameters to identify patients with significant CAD. Sabatino et al. [24] further extended the previous results of Edwards et al.’s study and found that GWI, GCW and GWE were significantly reduced in patients with critical CAD (stenosis >70%) as compared to the controls. The novelty of this study was the assessment of the regionalized MW indices. In particular, regional GWE had the highest diagnostic performance for predicting critical CAD (AUC = 0.920, *p* < 0.001), and this might solve one of the limitations of Edwards’ study, namely, the low specificity. Similar to the aforementioned studies, Zhang et al. [25] explored the value of global and regional MW indices in predicting high-risk, stable CAD in patients without wall-motion abnormalities and preserved LVEF. They demonstrated that GWI and GCW could predict high-risk, stable CAD at cutoff values of 1808 mmHg% and 2038 mmHg%, respectively, and that decreased GWI and GCW were independently related to high-risk, stable CAD in multivariable analyses. Compared to previous studies, the MW indices could not be proven as superior to GLS in identifying high-risk, stable CAD with statistical significance, and the limited sample size of the cohort included in this study could be a possible reason.

Another study [26] included patients with CAD (stenosis ≥ 50% in at least one major coronary artery), with heart failure (mid-range or reduced LVEF), without heart failure (preserved LVEF) and controls (healthy individuals). The CAD patients were divided into hypertension and no-hypertension subgroups. In this study, MW was more predictive for assessing LV function as compared to LVEF and GLS. The GWI and GCW values were decreased in CAD patients with heart failure and increased in the subgroup of CAD patients with preserved EF and hypertension subgroup vs. the controls. In hypertensive patients, LV must spend more energy to eject blood against an increased afterload, and this could explain the increased values in the hypertension subgroups. GWW was increased and GWE was decreased in all CAD subgroups.

When comparing the resting MW indices with stress myocardial perfusion (assessed by coronary-computed tomography angiography and dynamic-stress-computed tomography myocardial perfusion imaging) in patients with angina and non-obstructive CAD (lumen stenosis < 50%), the potential advantages over LVEF have been demonstrated [27]. Impaired stress myocardial perfusion in patients with non-obstructive CAD may be a consequence of coronary microvascular dysfunction. GLS, GCW, GWI and GWE were reduced and GWW was increased in patients with reduced stress perfusion, suggesting that these parameters could detect myocardial ischemia earlier and more accurately than LVEF. In the multivariable logistic regression, GWI and GWE were independently associated with reduced global-stress myocardial perfusion, while GLS was not. Among the analyzed variables, GWE had the highest AUC value (AUC = 0.858, *p* < 0.05), with an optimal cutoff value of 95% (specificity 90%, sensitivity 70%), which demonstrated that it is the most powerful parameter for detecting reduced global stress myocardial perfusion.

Recently, Zhou et al. [28] proposed a new method of predicting severe CAD (stenosis ≥ 50% in the left main coronary artery and ≥70% in at least one major coronary artery), a method named “positive region identification” according to the assignment of segments of the coronary artery territories and the values of the myocardial work segments in the bull’s-eye diagram of myocardial work in the LV. Using this method, the results of this study showed that, when compared with the regional values of GLS, regional GWI predicted severe CAD with higher sensitivity (95.2% vs. 70.2%) and similar specificity (97.5 vs. 91.1%) and performed better in accurately detecting the culprit coronary artery with severe stenosis (Table 2). The “positive region identification” method proposed in this study performed better in predicting severe stenosis in the culprit coronary artery as compared to the traditional method (regional average values in the anterior, lateral and inferior wall segments of the bull’s-eye plot, corresponding to the left anterior descending artery, left circumflex and right coronary artery); therefore, it may improve the accuracy of diagnosis and could have a strong clinical practicability.

In summary, the MW parameters could provide incremental diagnostic information for identifying patients with chronic CAD who may benefit from early therapeutic strategies. Further studies with large-scale and multicenter samples should be performed to improve the diagnostic and prognostic value of MW in stable coronary heart disease.

### 5.2. Acute Coronary Syndromes

Acute coronary syndromes (ACS) represent a range of conditions characterized by a sudden, reduced blood flow to the heart and are often the first clinical manifestation of cardiovascular diseases. These conditions include unstable angina, which is when blood flow is decreased but is not severe enough to produce myocyte death, and myocardial infarction, which is characterized by a partially blocked coronary artery; a transient, complete block of a coronary artery (non-STEMI); or a total blockage of a coronary artery (STEMI), resulting in cell injury or the necrosis of part of the myocardium [29]. In the last 40 years, major progress has been made in the management and treatment of ACS, and the prognoses of patients have significantly improved. However, continuous efforts are still being made to predict the outcome of patients with ACS [30]. LV dysfunction is a key prognostic factor in patients presenting ACS. Current guidelines recommend routine echocardiography before hospital discharge to assess LV, right ventricle and valvular function that may influence outcomes in ACS survivors [31]. Several studies evaluated the usefulness of MW in patients with ACS.

#### 5.2.1. Non-ST-Segment Elevation Myocardial Infarction

The changes in myocardial function were evaluated through MW assessment 1 day before and 1 month after percutaneous revascularization in 33 non-ST-segment elevation myocardial infarction (non-STEMI) patients as compared to 30 healthy subjects. Compared to the controls, the GLS, GWI, GCW and GWE values were decreased, while the GWW value was increased in non-STEMI patients 1 day before and 1 month after revascularization. The difference was statistically significant (*p* < 0.05), suggesting that the LV myocardial function was impaired. In the group treated by percutaneous coronary intervention, the values of GLS, GWI and GCW increased after 1 month as compared to those obtained 1 day before revascularization, showing that the LV systolic function was improved, while other echocardiographic parameters were not significantly different between the two groups [32]. Follow-up is necessary after revascularization to observe the value of MW and to estimate long-term prognosis.

Severe coronary artery stenosis leads to a decreased blood flow to the myocytes and to regional myocardial stunning. Hence, defining and quantifying LV risk areas is crucial for early interventions that may reduce mortality. Quin et al. [33] showed that patients with significant coronary artery stenosis had lower global and regional values of GLS, GWI, GCW and GWE and higher values of GWW as compared to patients without significant coronary artery stenosis. Regional GLS, GWI, GCW and GWE were significantly worse in territories of total coronary artery occlusion. The best parameter for predicting significant coronary artery stenosis was regional GWE, with a cut-off value of 96% (AUC = 0.80, sensitivity 73%, specificity 70% and *p* < 0.001).

Approximately 30% of patients with non-STEMI present acute coronary occlusion (ACO) that does not develop ST-segment elevation on the ECG [34]. Boe et al. [35] aimed to investigate the ability of the MW index to identify patients with ACO. The reduced MW index in ≥ 4 adjacent segments had good sensitivity and specificity for identifying ACO and was superior to other echocardiographic parameters. The cutoff value for GWI to identify segmental systolic dysfunction was <1700 mmHg%. Alterations in the loading conditions, such as high systolic blood pressure, lead to a decrease in the systolic shortening of a segment, which may be falsely interpreted as dysfunctional. The logistic regression analysis of this study demonstrated that an elevated systolic blood pressure decreased the ability of strain analysis to identify patients with ACO, while GWI was able to correct the falsely interpreted reduction in systolic function based on strain analysis.

#### 5.2.2. ST-Segment Elevation Myocardial Infarction

Acute ST-segment elevation myocardial infarction (STEMI) survivors are at high risk for future cardiovascular events, including recurrent myocardial infarction, arrhythmias, heart failure and death; thus, more knowledge is needed regarding these patients’ prognosis after treatment with STEMI in daily clinical practice.

Butcher et al. [36] evaluated the prognostic value of MW indices in patients with STEMI and reduced LVEF. They found that higher values of GWI were associated with a greater probability of LVEF normalization after 6 months of follow-up, while lower values of GWI (<750 mmHg%) were independently associated with all-cause mortality, giving additional information that could be used to detect patients who may benefit from prompt initiation of therapy.

In patients with anterior STEMI treated by percutaneous coronary intervention, GCW was the best parameter for predicting segmental and global LV recovery and was more severely impaired in patients with in-hospital complications (defined as reinfarction, heart failure, LV thrombus and death) [37].

Mahdiudi et al. [38] demonstrated in their study that the GWE values in patients with STEMI treated by percutaneous intervention were lower compared with patients who have cardiovascular risk factors and normal controls. Consistent with these findings, Lustosa et al. [39] demonstrated that lower values of GWE (<86%), measured by transthoracic echocardiography within 48 h of admission in patients with STEMI, were associated with higher rates of all-cause mortality. Furthermore, it had an incremental prognostic value over baseline clinical characteristics, such as LVEF and GLS. Similar to the results of Lustosa et al., GWE <91% was independently associated with a higher risk of major events (unplanned coronary revascularization, hearth failure and cardiovascular death), in a study by Coisne et al. [40] that explored the prognostic value of MW 1 month after an acute myocardial infarction (Table 3). The slight difference in the GWE thresholds between the two studies may be due to the time when the GWE was analyzed (48 h vs. 1 month) and to medical therapy optimization. Another difference was that Coisne et al. included both patients with non-STEMI and STEMI in their study. Ischemia after STEMI leads to reduced adenosine triphosphate formation, changes in myocardial metabolism, LV contractility dysfunction and a decrease in regional longitudinal strain, which can all lead to a decrease in LV myocardial efficiency [38].

An important risk factor for the development of heart failure and all-cause mortality in patients with STEMI is LV remodeling, which is caused by microvascular obstruction, inflammation and infarct size, and is defined as an increase in LVEDV ≥20% from the baseline [41]. It was demonstrated that GWI, GCW and GWE were reduced and GWW was increased in patients with LV remodeling 3 months after STEMI as compared to patients without LV remodeling. Interestingly, a minority of patients without remodeling also showed some degree of impaired MW, which may be related to further reverse remodeling at longer-term follow-ups [42].

Another study that aimed to evaluate the role of MW in patients with STEMI from the baseline to the 3-month follow-up found that the values of GWI, GCW and GWE were significantly improved at the follow-up, which may reflect the myocardial stunning (delayed myocardial function recovery after reperfusion in patients with STEMI), while GWW did not change between the baseline and the 3-month follow-up, which may reflect the development of non-viable, irreversible scar tissue [43].

Even after successful revascularization of the obstructed coronary artery, there is a relatively high incidence of coronary microvascular dysfunction that may affect outcomes in STEMI patients. Jin et al. [44] aimed to explore the role of MW in identifying impaired microvascular perfusion in patients with STEMI within 48 h after PCI and found that GWI, GCW, GWE and GLS were significantly reduced in the impaired microvascular perfusion group as compared to the normal microvascular perfusion group. In this study, GWI was the only independent predictor for impaired microvascular perfusion among the MW parameters, with a cutoff value of 1145 mmHg% (sensitivity, 86.8%, specificity, 53.7%). Several studies demonstrated the value of MW indices in predicting CAD (Table 4).

A recently published meta-analysis aimed to compare the diagnostic accuracy of MW parameters in predicting CAD. Five studies, which included a total of 501 patients, were evaluated. The results showed that GCW had the highest diagnostic accuracy among all MW indices in the prediction of CAD (AUC = 0.86), with an excellent reproducibility [45].

In conclusion, the evaluation of MW could be a promising tool in clinical practice for assessing the necessity of early revascularization and predicting the outcomes of patients with CAD.

## 6. Diabetes Mellitus

Heart failure is a major health concern, leading to frequent hospitalizations (with increasing prevalence over 65 years of age), high mortality and economic costs [46]. Approximately 24% of overall heart failure patients and 40% of hospitalized heart failure patients have DM. These patients have increased cardiovascular mortalities and worse outcomes [47]. Through the pathophysiologic mechanisms underlying diabetes-related heart failure, we mention microvascular dysfunction (microvascular remodeling that causes impaired production of nitric oxide with consequent endothelial dysfunction), metabolic alterations (insulin resistance that leads to a high release of free fatty acids and thereby to a dysfunction of myocardial mitochondria, resulting in increased oxygen consumption and, therefore, reduced myocardial efficiency), functional alterations (perturbations in calcium homeostasis leading to diastolic dysfunction) and neurohormonal abnormalities (increased activation of the renin-angiotensin-aldosterone system favoring cardiac fibrosis) [48]. Cardiac function impairments in patients with DM can occur early when clinical symptoms of heart failure are not obvious; therefore, the identification of subclinical LV dysfunction in the first stages of the disease is essential for the prevention and therapeutic management of patients with DM. Because it takes both afterload and strain measurements in its analysis, MW is more accurate than conventional parameters for evaluating LV systolic function. Although MW has shown great potential for several diseases, few studies have evaluated this new echocardiographic parameter in patients with DM.

Liao L et al. [49] studied the role of MW in patients with type 2 DM, excluding those with heart failure, coronary artery disease, atrial fibrillation, hypertension with poor blood pressure control or valvular disease. They demonstrated that, compared to the healthy controls, GWI was significantly decreased and GWW was significantly increased in these patients with no difference in GCW, leading to a decrease in GWE and indicating an impairment in myocardial function, while LVEF was still preserved. GLS was decreased in the diabetic group, but it was still dependent on loading conditions; therefore, MW could be a better method for identifying myocardial impairments in patients with type 2 DM. The differences and similarities between the global longitudinal strain and myocardial work are highlighted in Table 5. Consistent with the previous study, Huang et al. [50] showed that GLS and GWI were decreased in patients with type 2 DM as compared to the controls. The difference was that GCW was also decreased, with no statistical difference in GWW and GWE, which may be related to the small sample size of the cohort. The novelty of this study was that they explored the risk factors for MW impairments and demonstrated that HbA1c was an independent risk factor for GWI, and that diabetes duration in association with HbA1c was an independent risk factor that affected GCW; these results may be related to the LV dysfunction caused by a long-term hyperglycemic environment. Another study, consistent with the previous mentioned studies, found that GLS, GCW, GWI, and GWE were significantly reduced in patients with type 2 DM (without any history of cardiovascular diseases) while GWW was significantly increased, indicating myocardial dysfunction although LVEF was normal [51].

Interestingly, Tadic et al. [53] found that GCW was higher in hypertensive patients as compared to the controls but was even higher in hypertensive patients with both hypertension and DM, findings that could be related to pulse pressure. Type 2 DM is related to arteriosclerosis, which may lead to an elevation in pulse pressure and an increase in LV afterload [54,55].

Cao et al. [56] bring an element of novelty in their study that explored the LV function in patients with type 2 DM by the MW technique. They found that peak strain dispersion (PSD), a parameter assessed by 2D speckle tracking echocardiography that reflects the heterogeneity of contraction between LV myocardial segments, was positively correlated with GWW and negatively correlated with GWE. Increased values of PSD and GWW and decreased values of GWE with no significant differences in GWI and GCW were found in the type 2 DM group as compared to the controls. Early in the development of diabetes, GWI and GCW may be normal due to the compensation mechanisms. However, some myocardial segments of the LV may begin to relax before the end of systole while other segments continue to contract at the beginning of the diastole, leading to an asynchronism of the myocardial deformation and therefore to an increased PSD. Uncoordinated myocardial strain may be one of the reasons that explain the increased values of GWW and decreased values of GWE, indicating that these two parameters derived from MW might be more sensitive for the early identification of myocardial dysfunction in patients with type 2 DM.

Interestingly, MW was used to evaluate the effect of antidiabetic drugs on the LV function. Ikonomidis et al. [57] showed that patients had improved values of GWI and GCW and a reduction in GWW after twelve months of treatment with a combination of a sodium-glucose cotransporter-2 inhibitor (empagliflozin) and a glucagon-like peptide-1 receptor agonist (liraglutide) as compared to those treated only with insulin or empagliflozin.

Due to the global rise in the prevalence of DM in recent years, it is crucial to prioritize the early detection of myocardial dysfunction because it could play a significant role in the early treatment, follow-up, risk stratification and improved prognosis of patients with DM.

## 7. Other Clinical Implications of Myocardial Work

This novel imaging tool called MW has been in the spotlights of several research studies, and the clinical implications of this method have been expanding in the last few years. Among other cardiac conditions where the usefulness of MW has been demonstrated, we mentioned arterial hypertension [53,58,59,60,61,62], dilated cardiomyopathy [63,64], aortic stenosis [65,66,67], cancer therapy-related cardiotoxicity [68], heart failure [69,70,71,72], cardiac resynchronization therapy [73,74,75,76] and cardiac amyloidosis [77]. MW plays an important role not only in the cardiology field, but also in other pathologies that may induce LV dysfunction, such as chronic kidney disease [78,79], systemic lupus erythematosus [80] and COVID-19 infection [81].

## 8. Limitations of the Method and Future Directions

MW analysis is dependent on adequate two-dimensional image acquisition. Poor image quality that does not allow correct endocardial border delineation limits speckle tracking and myocardial work analysis [11]. The optimization of frame rates (typically exceeding 40 frames/s) is necessary for speckle tracking analysis, and the results may be influenced by the patient’s chest wall conformation. For instance, myocardial strain parameters may be impaired in individuals with concave-shaped chest walls or even minor pectus excavatum due to compressive effects, even in the absence of intrinsic myocardial dysfunction [82]. An accurate brachial cuff pressure is required for analysis and should be performed in the position of echocardiographic measurements at the beginning of the procedure to ensure that the blood pressure accurately correlates with the afterload of LV [8]. It is assumed that the aortic systolic pressure is equal to the LV systolic pressure in the absence of an LV outflow tract and aortic valve gradients. Consequently, patients with aortic stenosis or a fixed LV outflow tract were excluded from the original validation studies. However, the estimation of the LV afterloads in patients with aortic stenosis was recently investigated in some studies, and a new method that estimates the LV peak pressure was obtained by adding the mean aortic transvalvular gradient to the aortic systolic pressure, and an excellent correlation was observed between this method of performing MW and invasive measurements [64,65]. End-diastolic LV pressure, which reflects the preload, is not involved in the protocol of MW analysis, which considers only the LV work during systole and the afterload. MW analysis takes for granted the fact that the LV end-diastolic pressure is very low (2–3 mmHg) in normal hearts and does not affect the pressure-strain loop area [12]. Nevertheless, preload should be considered in future MW methodology to include all the patients suffering from volume overload conditions. Anatomic variations, such as wall thickness, left ventricle radius or curvature, are not accounted for in the protocol of MW analysis, but they may alter the wall stress applied on the segments and, consequently, MW [7]. The analysis of MW assumes that the LV wall thickness is the same across all myocardial segments, but regional hypertrophy can impact strain and consequently affect myocardial work as well [83]. Further studies that include all myocardial layers are needed to eliminate any false results coming from geometric presumptions. Also, the method assumes that the LV pressure is uniformly distributed throughout the entire LV wall, but, for example, in conditions as left bundle branch block, the activation of the LV free wall is delayed as compared to the septal wall, which leads to additional myocardial work [84]. Another limitation is that the MW utilizes only longitudinal strain and does not consider the work associated with circumferential and radial lengthening and shortening. However, these additional factors also play a significant role in LV contractility [7]. The presence of atrial fibrillation and other abnormal heart rhythms, especially when accompanied by excessive heart rate variability, can pose challenges to obtaining strain traces and accurately estimating MW [8]. Finally, to date, only one system (General Electric machines) provides the software for calculating myocardial work, limiting the applicability of this method.

## 9. Conclusions

The non-invasive assessment of MW, combining GLS data using two-dimensional speckle tracking echocardiography with the non-invasive measurement of systolic blood pressure, provides information about myocardial mechanics and energetics. MW shows promise as an innovative tool for assessing LV performance. It has the potential to distinguish between reduced LV systolic function caused by increased afterload and reduced myocardial contractility. The enhanced identification of subclinical cardiac dysfunction can serve as a valuable indicator of disease, leading to a deeper understanding of the underlying mechanisms of cardiac conditions. This improved detection not only aids in identifying potential therapeutic targets but also provides opportunities for early diagnosis and predicting outcomes. Non-invasive MW assessment remains an open door for clinical research intended to further investigate its role and applicability before it can be widely used in clinical practice.

## Figures and Tables

**Table 1:** Normal ranges of myocardial work parameters according to age and gender in four different studies.

Study	Age (Years)	Gender	GWI, mmHg%	GCW, mmHg%	GWW, mmHg%	GWE, %
EACVI NORRE study [13] *	20–40	Male	1758 ± 270	2186 ± 240	99 (68–144.5)	95 (93–97)
		Female	1800 ± 251	2109 ± 289	90 (48–145)	95 (94–97)
	40–60	Male	1900 ± 317	2267 ± 327	89 ± (58–122.5)	96 (95–97)
		Female	2027 ± 341	2329 ± 365	76 (51–118)	96 (95–97)
	≥60	Male	1866 ± 286	2226 ± 328	85 (49–129)	96 (94–97)
		Female	2002 ± 270	2338 ± 386	90 (48–145)	95 (94–97)
STAAB [14] **	≤45	Male	2141 (2099–2183)	2366 (2330–2402)	73 (70–76)	96 (96–96)
		Female	2206 (2168–2245)			
	>45	Male	2187 (2149–2224)	2457 (2428–2486)	-	-
		Female	2252 (2220–2284)			
Galli et al. [15] *	≤25	Male	1912 ± 217	2231 ± 217	93 (64–115)	95 (95–97)
		Female	1923 ± 276	2172 ± 238	114 (69–154)	95 (93–96)
	25–35	Male	1927 ± 255	2179 ± 200	72 (52–120)	96 (94–97)
		Female	1954 ± 109	2260 ± 267	93 (62–112)	95 (94–96)
	35–45	Male	1832 ± 252	2184 ± 250	111 (63–149)	94 (93–97)
		Female	2190 ± 206	2367 ± 207	71 (50–96)	97 (96–98)
	≥45	Male	1824 ± 196	2174 ± 145	81 (69–94)	96 (95–96)
		Female	2058 ± 275	2350 ± 306	92 (59–132)	96 (94–97)
CCHS [16] *	32–57	Male	2062 ± 269	2229 ± 275	60 (42–84)	97 (96–98)
		Female	2155 ± 275	2283 ± 286	68 (49–93)	97 (95–97)

* mean ± SD or median (IQR); ** mean (95% CI); GWI = global work index; GCW = global constructive work; GWW = global wasted work; GWE = global work efficiency; LV = left ventricle; EACVI NORRE = European Association of Cardiovascular Imaging Normal Reference Ranges for Echocardiography; STAAB = The Characteristics and Course of Heart Failure STAges A/B and Determinants of Progression; CCHS = Copenhagen City Heart Study; SD = standard deviation; IQR = inter-quartile range; CI = confidence interval.

**Table 2:** Cut-off values of GLS_R_ and GWI_R_ assessed by “positive region identification” [28].

PARAMETER	LAD	LCX
GLS_R_ (%)	−18.6	−16.9
GWI_R_ (mmHg%)	1814	1771

LAD = left anterior descendent artery; LCX = left circumflex artery; RCA = right coronary artery, GLSR = regional global longitudinal strain; GWIR = regional global work index.

**Table 3:** Association of GWE and GWI with adverse outcomes in patients with ACS in different studies.

Myocardial Work Parameter	Value	Role
GWE	<86%	Independent association with all-cause mortality in patients with STEMI (HR 3.167 [95% CI, 1.679–5.927]; *p* < 0.001) [39].
GWE	<91%	Independent association with higher risk for major events in patients after an acute myocardial infarction (HR 2.94 [95% CI, 1.36–6.35]; *p* < 0.041) [40].
GWI	<750 mmHg%	Independent association with all-cause mortality in patients with STEMI (HR 3.85 [95% CI, 1.94–7.67]; *p* < 0.0001) [36].

GWE = global work efficiency; GWI = global work index; STEMI = ST-elevation myocardial infarction; HR = hazard ratio; CI = confidence interval.

**Table 4:** Cutoff values of the myocardial work indices in different studies.

Myocardial Work Parameter	Cutt-Off Value	Sensitivity (%)	Specificity (%)	Role
GWE	78%	90.5	85.7	To predict critical coronary artery stenosis [24]
GWE	95%	70	90	To detect reduced global stress myocardial perfusion in patients with angina and non-obstructive coronary artery disease [27]
Regional GWE	96%	73	70	To predict obstructive coronary artery stenosis [33]
GWI	1145 mmHg%	86.8	53.7	To predict microvascular perfusion impairments in patients with STEMI [44]
GWI	1810 mmHg%	92	51	To predict significant coronary artery disease [23]
GWI	1808 mmHg%	52.6	87.8	To predict high-risk, stable coronary artery disease [25]
GCW	2308 mmHg%	80.7	64.9	To predict high-risk, stable coronary artery disease [25]

GWE = global work efficiency; GWI = global work index; GCW = global constructive work; STEMI = ST-elevation myocardial infarction.

**Table 5:** Differences and similarities between the global longitudinal strain and myocardial work [8,52].

**Differences**	**Definition and measurement**	**GLS**	Measures the percentage change in length of the myocardium along its longitudinal axis during the cardiac cycle and quantifies the deformation of the myocardium
		**MW**	A measure of the energy expended by the myocardium during the cardiac cycle and is derived from the pressure-strain loop.
	**Assessment**	**GLS**	Primarily focuses on myocardial deformation and provides information about strain. It is a marker of contractile function.
		**MW**	Focuses on the mechanical work performed by the heart, providing insights into the energy expended by the myocardium during the cardiac cycle.
	**Parameters**	**GLS**	Is a single parameter and represents the global longitudinal deformation of the left ventricle.
		**MW**	Is characterized by four parameters (GWI, GCW, GWW and GWE) that describe different aspects of myocardial performance.
	**Load** **dependency**	**GLS**	Can be influenced by changes in preload and afterload.
		**MW**	Incorporates deformation and load into its analysis.
**Similarities**	**Assessment**	Both GLS and MW are assessed using speckle tracking echocardiography.	
	**Accurate** **measurements**	Dependency on frame rate, good imaging quality and heart rate variability.	
	**Visualization**	Bull’s-eye plots are commonly used to visualize their results.	
	**Clinical value**	Both GLS and MW play a crucial role in the assessment of LV function. Both have demonstrated prognostic value in various heart diseases.	

GLS = global longitudinal strain; MW = myocardial work; GWI = global work index; GCW = global constructive work; GWW = global wasted work; GWE = global work efficiency; LV = left ventricle.

## Data Availability

No new data were created or analyzed in this study. Data sharing is not applicable to this article.
